# Deep convolution neural network for screening carotid calcification in dental panoramic radiographs

**DOI:** 10.1371/journal.pdig.0000081

**Published:** 2023-04-12

**Authors:** Moshe Amitay, Zohar Barnett-Itzhaki, Shiran Sudri, Chana Drori, Tamar Wase, Imad Abu-El-Naaj, Millie Kaplan Ben-Ari, Merton Rieck, Yossi Avni, Gil Pogozelich, Ervin Weiss, Morris Mosseri

**Affiliations:** 1 ODMachine Ltd., Herzliya, Israel; 2 Bioinformatic Department, Jerusalem College of Technology, Jerusalem 9372115, Israel; 3 Faculty of Engineering, Ruppin Academic Center, Emek Hefer, Israel; 4 Ruppin Research Group in Environmental and Social Sustainability, Ruppin Academic Center, Israel; 5 Department of Oral and Maxillofacial Surgery, Baruch Padeh Medical Center, Poriya, affiliated with Azrieli Faculty of medicine, Bar Ilan University, Israel; 6 Azrieli Faculty of Medicine, Bar Ilan University, Safed, Israel; 7 Goldschleger School of Dental Medicine, Sackler Faculty of Medicine, Tel Aviv University, Tel Aviv, Israel; 8 Sackler Faculty of Medicine, Tel Aviv University; University of Toronto, CANADA

## Abstract

Ischemic stroke, a leading global cause of death and disability, is commonly caused by carotid arteries atherosclerosis. Carotid artery calcification (CAC) is a well-known marker of atherosclerosis. Such calcifications are classically detected by ultrasound screening. In recent years it was shown that these calcifications can also be inferred from routine panoramic dental radiographs. In this work, we focused on panoramic dental radiographs taken from 500 patients, manually labelling each of the patients’ sides (each radiograph was treated as two sides), which were used to develop an artificial intelligence (AI)-based algorithm to automatically detect carotid calcifications. The algorithm uses deep learning convolutional neural networks (CNN), with transfer learning (TL) approach that achieved true labels for each corner, and reached a sensitivity (recall) of 0.82 and a specificity of 0.97 for individual arteries, and a recall of 0.87 and specificity of 0.97 for individual patients. Applying and integrating the algorithm in healthcare units and dental clinics has the potential of reducing stroke events and their mortality and morbidity consequences.

## Author summary

Stroke is a leading global cause of death and disability. One major cause of stroke is carotid arteries atherosclerosis. Carotid artery calcification (CAC) is a well-known marker of atherosclerosis. Traditional approaches for CAC detection are doppler ultrasound screening and angiography computerized tomography (CT), medical procedures that incur financial expenses, and are time consuming and discomforting to the patient. Of note, angiography CT involves the injection of contrast material and exposure to X-ray ionizing irradiation. In recent years researchers have shown that CAC can also be detected by analyzing routine panoramic dental radiographs, a non-invasive, cheap and easily accessible procedure. This study takes us one step further, in developing artificial intelligence (AI)-based algorithms trained to detect such calcifications in panoramic dental radiographs. The models developed are based on deep learning convolutional neural networks and transfer learning, that enable an accurate automated detection of carotid calcifications, with a recall of 0.82 and a specificity of 0.97. Statistical approaches for assessing predictions per individual (*i*.*e*.: predicting the risk of calcification in at least one artery) were developed, showing a recall of 0.87 and specificity of 0.97. Applying and integrating this approach in healthcare units may significantly contribute to identifying at-risk patients.

## Introduction

Stroke is the third leading cause of death and the leading cause of disability in the Western world. Ischemic stroke is caused by carotid arteries atherosclerosis, small intracranial vessel disease or emboli from the heart and aorta [1,2]. The lifelong risk of stroke in adult men and women (age 25 and older) is about 25 percent [3]. Ten percent of strokes are caused by intracerebral hemorrhage and 87% of all strokes are ischemic [2]. Several studies showed that patients aged 60–96 with carotid artery calcification (CAC) found in panoramic radiograph are 2.4 fold more likely to suffer from vascular events, including stroke and/or ischemic heart diseases [4,5]. Although evidence for the predictive value of CAC is still variable, as reviewed by Lim *et al*, it is established as useful for identifying at risk patients and referral for further evaluation [6].

Standard tests for detecting CAC are doppler ultrasound (US) and angiography computerized tomography (CT). However, there is evidence that calcification can be detected in panoramic dental X-rays (dental radiographs) [7–11]. These X-rays are routinely performed in daily practice by dentists and oral and maxillofacial surgeons. A panoramic radiograph is a two-dimensional interpretation of tomographic images of curved anatomic structures. Panoramic radiography images serve as a diagnostic tool, and the image encompasses the teeth, the maxillary and mandibular bones, temporomandibular joints, and the maxillary sinus. Nonetheless, most dental professionals, dentists as well as specialists, are not trained for detecting and diagnosing CAC in panoramic X rays. Several studies focused on evaluating the ability of panoramic radiographs to detect CAC [5] and showed the potential in the use of panoramic radiographs to help identify at-risk patients who require further evaluation [8]. Recent meta-analyses of these studies revealed that the level of agreement between panoramic radiography and the above standard methods is 50% [12]. However, even with this limitation, panoramic radiography is more prevalent by far than US or coronary angiography (CAG). The panoramic radiograph has a sensitivity of 66.6% and a positive predictive value of 45% for detecting carotid artery calcifications in patients whose angiograms confirmed coronary artery disease [13]. Therefore, panoramic radiography may play an important role in the screening and detection of non-symptomatic CAC patients in the population.

Deep neural networks are a branch of machine learning (ML) and artificial intelligence (AI). Deep learning algorithms use architectures that are composed of multiple artificial neurons to form neural networks (NN) that can predict a value/class for new samples. These architectures were developed to tackle complex challenges such as speech recognition, natural language processing, image classification and object recognition [14]. Deep learning architectures and algorithms use multilayer artificial neural network (NN) architecture. A major class of deep learning algorithms is the convolutional neural networks (CNN), that are widely used for image classification [15]. In order to cope with potential biases and to produce the most efficient networks, it may be advisable to optimize the convolution neural networks [16]. Major challenges in the development of an efficient CNN classifier are the requirement for large numbers of training samples (usually >1,000 for each class), and a long and comprehensive process of model training. In order to cope with these challenges, the transfer learning (TL) approach was developed. In this approach, the CNN training is not created from scratch, but uses an existing pre-trained model as a starting point [17,18]. The pre-trained model was previously trained on a different task using huge amounts of data.

CNN and TL have been widely used in the prediction of medical conditions using different techniques (CT, MRI, panoramic images)–for example: identification of prostate cancer [19]; prediction of bladder cancer treatment response in CT [20]; detection of maxillary sinusitis on panoramic radiographs [21]; screening for osteoporosis in dental panoramic radiographs (DPR) [22]; cardiac cine segmentation [23] and even COVID-19 detection from chest CT-scans [24]. Furthermore, Kats *et al*. have recently shown the potential of applying CNN to detect CAC, using Faster Region-based Convolutional Neural Network (FR-CNN) [25] on a modest set of 65 DPRs reaching a F1 score of 0.77 [26].

In this study we aimed to develop and evaluate a robust image classifier for screening carotid calcification (CAC) in standard (DPR) images using a relatively large cohort of hundreds of DPRs, utilizing advanced approaches. The benchmark in this study was the human annotations of CAC in a panoramic radiograph. We trained and tested a convolutional neural network (CNN) based on transfer learning for CAC detection of a single carotid (one side of the image) and then calculated the performance of a full panoramic radiography images. Our algorithm reached good performances of recall of 0.87 and specificity of 0.97.

## Materials and methods

### Study population and ethical approval

#### Ethics statement

This study was conducted in accordance with the Declaration of Helsinki, seventh revision (2013), national and institutional standards, and approved by the Poriya Medical Center Institutional Review Board (IRB approval # POR-0008-21). As a retrospective trial using anonymized data, formal consent was not required. Patient records/information were fully anonymized and de-identified prior to receiving the data for analysis.

#### Study population

In total, the study included 500 patients who visited the oral and maxillofacial department at the Poriya Medical Center between 2016 and 2021 and met the following criteria: Inclusion criteria were as follows: (a) patients who were 40 years old and older, and (b) had a panoramic radiograph encompassing both jaws (upper and lower), the hyoid bone, and the fourth upper cervical spine vertebrae. Exclusion criteria were as follows: (a) low quality panoramic radiographs with trimmed corners and/or blurred and spread spinals; (b) treatment with coumadin (warfarin); (c) diagnosis of hypomagnesemia; and (d) diagnosis of hypercalcemia due to malignancy.

The following parameters were elicited anonymously from the patients’ medical files: age, sex, smoking history, alcohol and drug abuse, weight, height, and systemic medical history.

Determining whether the sample size is sufficient for this study was performed using random subsampling of the data and repeating the analysis, based on the approach suggested by Balki *et al*. [27]. See also S1 Text.

#### Panoramic radiographs

All the panoramic radiographs were performed on a Planmeca ProMax 3D (Planmeca Oy, Finland). The clinical files and panoramic radiographs were anonymized. Image view and analysis was performed on an Acer XB273K GP, 4K, 120HRZ, ‘Predator’, UHD IPS, G-SYNC, HDR400, 27 inches (Taipei, Taiwan), installed on an ASUS ROG computer, GT35 G35CZ-IL030D, I7-10700KF,1T M.2 NVME,16G, NVIDIA, RTX3070-8GL, (Taipei, Taiwan).

#### Image labeling

The panoramic images were labeled by two certified oral and maxillofacial physicians that were trained by an oral radiology specialist. Labeling was based on the location, texture, and morphologic features of stained areas in the images, as defined and described in previous works [10,28,29] and will be discussed later. There was a high agreement of 84% between the two physicians regarding the labels of a panoramic image. In case of disagreement, it was re-evaluated by a further two certified oral and maxillofacial physicians, in order to reach a consensus. The images were labeled to two classes, in which both sides (corners), the left carotid artery and the right carotid artery, were labelled individually (*i*.*e*. two labels for each panoramic image).

- Carotid calcification (CAC)—non-homogeneous irregular calcifications located adjacent to the C3-C4 intervertebral space (these characteristics differentiate CAC from other calcifications such as triticeous cartilage calcification [see S1 Fig and S2 Fig, depicting various examples of CACs and triticeous cartilage calcification]); and
- No carotid calcification (including “clean” images with no calcification and calcification from non-carotid sources such as triticeous cartilage calcification). Both sides (corners), the left carotid artery and the right carotid artery, were labelled individually (*i*.*e*. two labels for each panoramic image).

### Data preprocessing

The main location of CACs is adjacent to the C3-C4 spinals. We filtered out images with trimmed corners and/or with low quality corners such as very blurred and spread spinals in the areas of interest. The final dataset consisted of 480 clean and 179 CAC corners.

**Fig 1:**
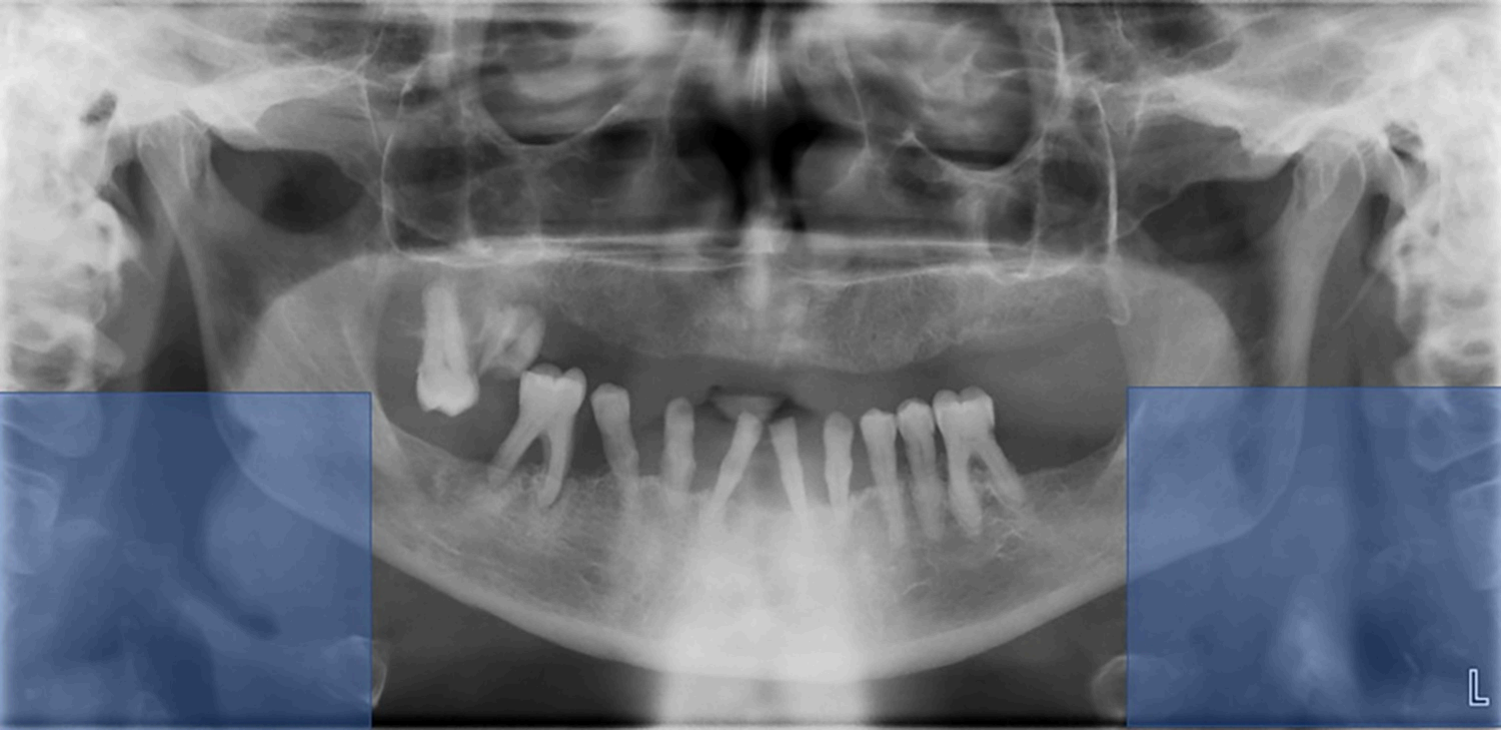
The region of interest is located near the C3-C4 spinals. Therefore, we cut out each of the lower corners of the panoramic image in 500 X 500 pixels size (depicted with light blue rectangles). This size ensured that the corners encompassed the calcification.

Since the location of the calcifications of the carotid artery is adjacent to the spinal cord, we cut out each of the lower corners of the panoramic image in 500 X 500 pixels size (depicted in Fig 1). Each corner was analyzed separately.

### Convolutional Neural Networks (CNN) and Transfer Learning (TL)

We trained CNN using a TL approach based on the pre-trained architecture: DenseNet169 [30], InceptionResNetV2 [31], and EfficientNetV2M [32], which are commonly used architectures that are considered as fast, efficient and with good performances. The same hyper parameters optimization procedures were applied for all networks. We applied image augmentation with operations such as width shift, zoom, rotation and shear. The unbalanced classes were treated in the training set by using different class weights. We used cross-entropy loss function (a detailed representation of the model architecture is depicted in S1 Table). Additionally, we applied InceptionResNetV2 followed by flatten layer and XGBoost classifier [33]. We carried out two training routines: an initial training of only the top layer, and a full model training. Of note, further structural CNN optimization of the other layers was made redundant due to the use of transfer learning, and therefore, we kept with the original CNN architecture. We used Adam optimizer [34] with 10^−3^ and 10^−5^ learning rates for the first and second training procedures respectively. The training was stopped when the validation loss didn’t decrease during 20 epochs. We used the Keras and TensorFlow libraries (version 2.10.0) [35].

### Algorithm evaluation

Due to the relatively small number of corners, we evaluated the algorithm performance using a 7-fold cross-validation approach. We created an imbalance in the folds of 1:5 CAC as a reasonable balance between “real-world” occurrence rates and the requirement to emphasize carotid calcification. In each fold we split the data into three sets: (a) test set–with 24 CAC corners and 120 clean ones, (b) validation set–with 24 CAC corners and 120 clean ones, and (c) training set—with 131 CAC corners and 240 clean ones. In order to rule out a potential bias in the demographic parameters between the training, validation, and the test sets (*i*.*e*. higher proportions of male participants in one set in comparison to another), Wilcoxon non-paired tests were used to compare the ages of the three sets while Chi-square tests were used to compare the other demographic parameters in each fold: sex, smoking, alcoholism, drug abuse, and history of cardiac infractions.

The performance of the network model after concatenation can be evaluated by determining statistical values (recall, specificity, precision, and accuracy) and the F1-score. The recall, known also as sensitivity reflects the positive proportion of correct recognition $\left(\frac{True\;positive}{True\;positive+False\;negative}\right)$, the specificity reflects the negative proportion of correct predictions $\left(\frac{True\;negative}{True\;negative+False\;positive}\right)$, the precision is the fraction of the correct predictions among the retrieved instances $\left(\frac{True\;positive}{True\;positive+False\;positive}\right)$. F1-score is an evaluation index which takes both precision and recall into account $\left(2*\frac{presicion*recall}{precision+recall}\right)$.

### Calculation of prediction per patient (two sides) from prediction of the individual sides

This analysis was meant to calculate the algorithms’ performances focusing on patients rather than on individual corners. The statistical calculations are based on the corner classification algorithms and their performances. As described before, each panoramic image (of a specific patient) has two corners. Therefore, there are three types of patients: (a) a patient with two clean corners (Clean-Clean–CC); (b) a patient with one clean corner and one carotid calcified corner (Calcified-Clean–MC); or (c) two carotid calcified corners (MM).

We used the performance on a single corner (presented in Table 1) to calculate:

- p_1_ = probability of predicting the actual calcified corners out of the true calcified corners (also known as the recall).
- 1-p_1_ = probability of mistakenly predicting clean corners out of the of true calcified corners.
- p_2_ = probability of predicting “clean” out of the true clean corners (also known as the specificity).
- 1-p_2_ = probability of predicting “calcified” out of the true clean corners.

These probabilities enable the calculation of the probabilities of each of the following scenarios related to the three patient types (See Table 1).

**Table 1:** Probability calculations of the three patient types.

*Actual* *Prediction*	MM	CM	CC
**Clean**	*(i)* (1-p_1_)^2^	*(iii)* p_2_(1-p_1_)	*(v)* p_2_^2^
**CAC**	*(ii)* 1-(1-p_1_)^2^	*(iv)* p_2_(p_1_-1) +1	*(vi)* 1- p_2_^2^

*(i)* The probability of predicting a MM patient (with both corners calcified) as “clean” is the probability of mistakenly predict “clean” on both sides of the MM patient.

*(ii)* The probability of predicting a MM patient (with both corners calcified) as CAC (with at least one calcified corner) is the complementary probability 1-the probability calculated in (a).

*(iii)* The probability of predicting a CM patient (with one clean corner and one calcified corner) as “clean” is the multiplication of the specificity (actual clean) by the complementary of the recall.

*(iv)* The probability of predicting a CM patient as CAC is the complementary probability: 1-the probability in (c). 1-p_2_(1-p_1_) = 1-p_2_+p_1_p_2_ = p_2_(p_1_-1) + 1.

*(v)* The probability of predicting a CC patient (with both clean corners) as “clean”, is the multiplication of p_2_ (specificity) of one corner by the specificity of the other corner.

*(vi)* The probability of predicting a CC patient (with both clean corners) as CAC, is the complementary probability 1-the probability calculated in (e).

## Results

500 patients participated in the cohort. The average age was 67.5±13.3 years, 56% were male and 44% were female. 19.7% of the patients were smokers, 40% had diabetes, 63.7% had hypertension and 41.6% had a history of cardiac infarction.

No bias was found in the demographic parameters when comparing the training, validation, and test sets in each of the 7-fold (p>0.05). Table 2 presents the prediction performance blind tests and shows that the best classifier, InceptionResNetV2, showed recall (sensitivity) of 82%, precision (prevalence of the real CACs in the predicted positives) of 84% and specificity of 97%.

**Table 2:** Performance of predicting CAC from panoramic images (7-folds CV).

	F1	Recall	Precision	Specificity	Accuracy
InceptionResNetV2 (minimum-maximum)	0.82± 0.05 (0.76–0.91)	0.82 ± 0.09 (0.67–0.92)	0.84± 0.07 (0.73–0.95)	0.97± 0.02 (0.93–0.99)	0.94± 0.01 (0.93–0.97)
InceptionResNetV2 + XGBoost	0.8 ± 0.1	0.68 ± 0.08	0.73 ± 0.05	0.91 ± 0.04	0.91 ± 0.03
DenseNet169	0.79 ± 0.05	0.8 ± 0.1	0.81 ± 0.09	0.96 ± 0.03	0.93 ± 0.02
EfficientNetV2M	0.79 ± 0.09	0.78 ± 0.1	0.8 ± 0.09	0.96 ± 0.2	0.93 ± 0.03

**Fig 2:**
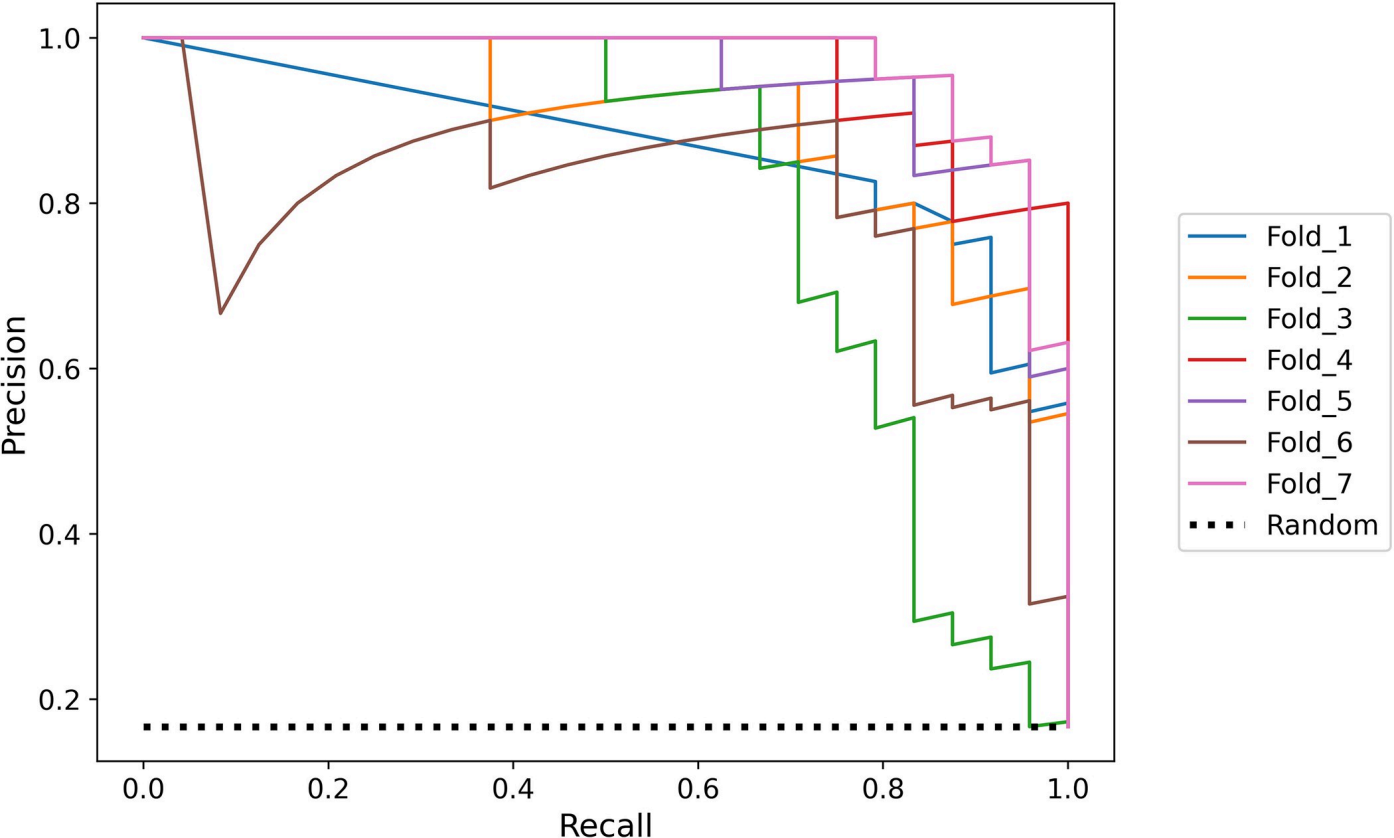
Recall-Precision (RP) curves of all the cross-validation folds (each fold is shown here in a different curve); the random classifier is depicted with a dashed line.

The Recall-Precision (RP) curve for all the cross-validation folds is presented in Fig 2. An RP curve is more informative than the usual ROC curve when the test is imbalanced and the performance on the minority class (*i*.*e*. the CAC) is more important. The curves show the trade-off between recall and precision of the 7-folds. It can be noticed that fold 3 slightly deviated from the rest of the folds. Thus, the actual performance may be somewhat better than the above. In addition, these curves show that we can achieve a higher recall value of 90% at the cost of decreasing the precision to 60%.

**Fig 3:**
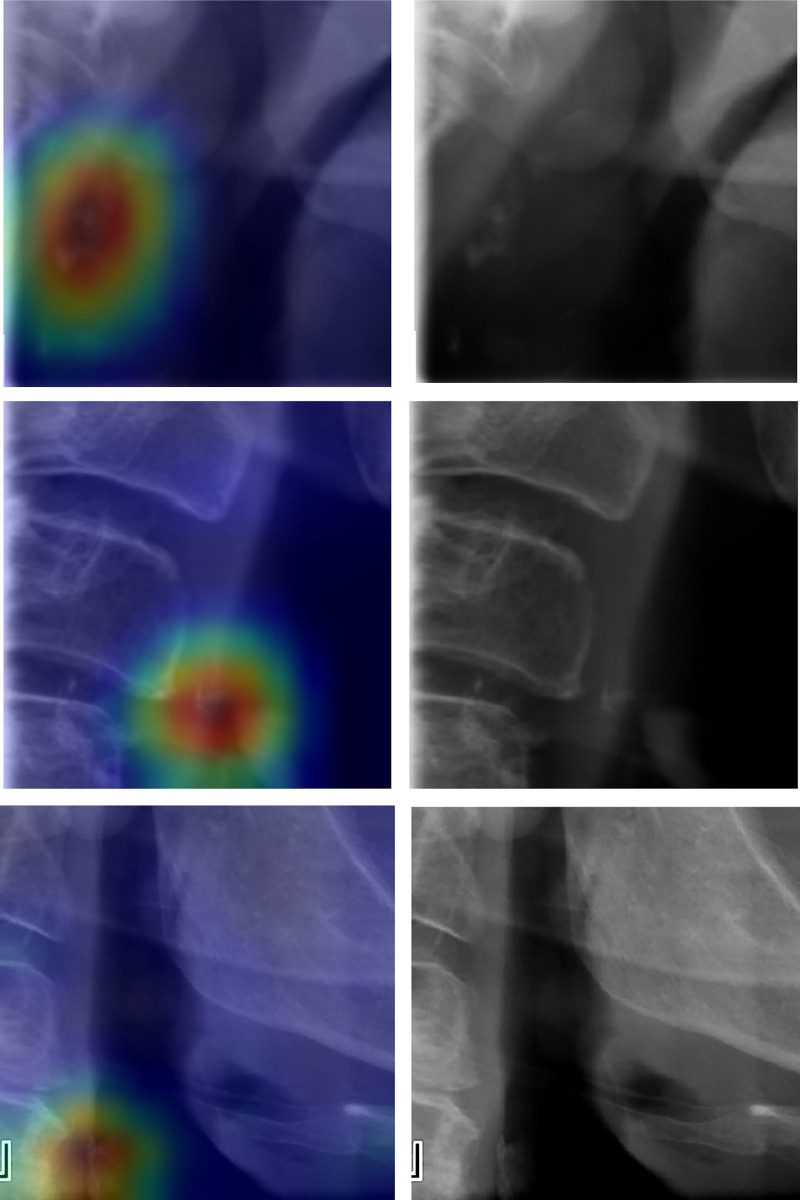
Gradient-weighted class activation mapping (Grad-CAM) of a correct prediction of CAC and “clean” classes. The colors indicate the region that has the greatest impact on the CAC prediction. The color range is presented below (ranging from red, representing the regions with the greatest impact on the CAC prediction, to blue, representing the regions with the lowest impact). It can be noticed that the classifier indeed focused on the region of the calcification signs. Other Grad-CAM of CAC and clean predications can be found in the S3 Fig and S4 Fig.

In addition, we evaluated the classifier by the determination of the specific image area that was important for class prediction. We employed the gradient-weighted class activation mapping technique (Grad-CAM) [36] to present the most significant regions for screening CAC in order to verify that the classifier indeed concentrated on the calcification areas when it predicted CAC. Fig 3. presents Grad-maps which highlight the important regions in the image for predicting both CAC and “clean”—thus providing visual explanations to the predictions. These maps show that the classifier indeed focused on the calcification areas of the CAC images. It can be noticed that the region of the calcification signs is the most significant for the CAC prediction. Other Grad-CAM images can be found in S3 Fig, S4 Fig and S5 Fig.

Although the total number of images was limited, we found that the sample size is sufficient for this study (See S1 Text and S1 Fig).

We used the p_1_ (recall—probability of predicting the actual calcified corners out of the true calcified corners and p_2_ (specificity—probability of predicting “clean” out of the true clean corners) that were calculated *per corner* (0.82, 0.93 respectively) as in Table 1 In order to calculate the performances *per patient*.

Assuming that a 1/3 of the CAC patients are MM and the other two thirds are CM [37], and that the ratio of CAC vs. clean is 1:5, the probabilities of predicting X or C on the three types of patients with the actual p_1_ and p_2_ from the performance on a single side is presented in Table 3. Assuming that the dataset consists of 144 patients: 8 MM, 16 CM and 120 CC we would get the confusion matrix presented in Table 3. Therefore, this table can be further used to calculate the performance per patient (see the methods section).

**Table 3:** The probabilities and quantities of prediction CAC or “Clean” on three types of patients based on the performance of a single side. The cells in the table consist of the probabilities and the patient numbers are presented in italic in parentheses.

	MM	CM	CC
**Clean**	0.03 *(0*.*26)*	0.17 *(2*.*79)*	0.94 *(112*.*91)*
**CAC**	0.97 *(7*.*74)*	0.83 *(13*.*21)*	0.06 *(7*.*09)*

Table 3 parameters were calculated using p_1_ = 0.82 and p_2_ = 0.97 from the single corner prediction. MM represents patients with both corners calcified; CM represents patients with one clean corner and one calcified corner; CC represents patients with both clean corners.

Assuming that the dataset consists of 144 patients as follows: 8 MM, 16 CM and 120 CC, the resulting calculated performance per patients is presented in Table 4: recall (sensitivity) of 0.87, precision of 0.75 and specificity of 0.97.

**Table 4:** Summary—performances ‘per corner ‘and ‘per patient’.

	Per Side	Per Patient
**Recall (sensitivity)**	0.82	0.87
**Specificity**	0.97	0.97
**Precision**	0.84	0.75

The increase in recall is due to higher probability of correctly predicting CAC of the MM patients compared to the prediction for an individual corner. The decrease in precision is due to the higher probability of mistakenly predicting CAC of the CC patients compared to the prediction for an individual corner. Since the goal is to detect the patients with CAC, we would rather raise the recall in trade of lowering the precision.

## Discussion

Prediction of stroke is still one of the major challenges in western medicine. Atherosclerosis of the carotid arteries is an important etiology for ischemic stroke. The main risk factors for atherosclerosis are hypertension, diabetes, hyperlipidemia, high cholesterol levels, smoking and obesity, all of which cause endothelial cell dysfunction. Atherosclerosis tends to calcify over the years. Therefore, carotid artery calcification is a manifestation of advanced atherosclerosis in the carotid arteries as well as a marker for atherosclerosis in other blood vessels, including coronary artery disease and peripheral vascular disease in the lower extremities. Early diagnosis of carotid arteries calcification (atherosclerosis) would prevent stroke by diagnosing, monitoring and treating carotid arteries stenoses as well as detecting and treating risk.

Carotid calcifications can be detected by performing a carotid ultrasound screening, but this is not a routine procedure, and is usually recommended only when a murmur is detected on auscultation or upon evidence of lower limb peripheral vascular disease, or in the presence of medical conditions that increase the risk of stroke. Periodic ultrasound screenings of the carotid arteries could detect carotid arteries atherosclerosis and calcification before the appearance of clinical manifestation; however, such a policy would involve a huge financial burden and is thus impractical. CT angiography is another test that detects atherosclerosis and calcification in carotid arteries. It involves the injection of contrast material and exposure to X-ray ionizing irradiation which, in addition to significant financial expenses, make this test inadequate for screening purposes. Panoramic dental X-rays may provide important information on carotid artery calcification [26,38,39]. They are performed routinely and the information on possible CAC can be retrieved without additional clinical test or procedure.

In this work we developed an AI-based algorithm that can efficiently diagnose calcified atherosclerosis in the carotid arteries, using routine panoramic dental X-rays images. Such diagnosis once available, should direct the treating physician to refer the patient for further evaluation and treatment of carotid artery narrowing, and indicates risk factors for atherosclerosis in various blood vessels, including those causing coronary artery disease and peripheral vascular disease of the lower limbs.

The first challenge in this study is the absence of a typical constant structure to the signs of calcification, *i*.*e*. there are no general characteristics of CAC that provide common range of shapes and orientations. Additionally, this region in the panoramic images contains background noise and other organs/bones, including the hyoid bone and various shapes of the spinal cord. One approach we used to cope with this challenge was through TL, that was successfully implemented in previous medical studies, including AI-based studies that analyzed panoramic radiographs [21,22].

An earlier study presented a computer-aided rule-based approach for detecting carotid calcification in panoramic radiographs using grayscale gradients and top-hat filters [40]. More recent studies have employed Faster Region-based Convolutional Neural Network (Faster R-CNN) [25] to detect carotid calcification in panoramic radiographs. Kats *et al*. [26] reported a sensitivity of 75%, specificity of 80%, and accuracy of 83%. Song *et al*. presented AI-based detection of three soft tissue diseases, including carotid artery calcification, using faster R-CNN on panoramic images, reporting a sensitivity of 77.4% and specificity of 71.4% for CAC detection [41].

Computer aided screening of calcification in radiological images is not specific only to the current challenge. There are other procedures in which it can be adopted, such as for detecting coronary calcification in intravascular Optical Coherence Tomography (OCT) and detecting calcifications in breast mammograms. Several studies aimed to computationally screen calcifications using AI and CNN approaches have been published: Li *et al*. used CNN to automate the segmentation and quantify coronary calcification in intravascular OCT images, reaching a F1 score of 0.96 [42]; Fuhrman *et al*. developed an algorithm based on both CNN Support Vector Machine (SVM) algorithm to classify coronary artery calcifications in low dose thoracic CT [43]. Other studies used a variety of deep neural network approaches based on CT images to predict different pathologies, such as transcatheter aortic valve replacement [44], chemotherapy response in breast cancer [45], quantitative assessment of liver trauma [46], and even the evaluation of complications associated with metastatic spine tumor surgery [47].

Panoramic radiographs are a routine part of oral and maxillofacial examinations. The high number of panoramic X rays performed routinely in dental clinics can provide an important and efficient source for the early detection of calcifications. Nevertheless, the inadequate training and awareness of dental personnel in detecting pathologies of the neck region, especially carotid artery calcifications, results in the ignoring of vast amounts of available information that has a high potential for the diagnosis, prevention, and monitoring of atherosclerotic changes in the carotid arteries. We believe that the current study lays the foundation for a valuable clinical tool aimed at providing health professionals with information for referring patients to an appropriate specialist. This novel clinical tool may be used on a wide basis in healthcare organizations, both dental and medical.

The present study has several limitations. Manual labeling (by a physician) is challenging: CACs can be confused with other soft tissue calcifications in the same radiologic region, such as the triticeous cartilage calcification. Finally, it is not possible to make a conclusive diagnosis without doppler ultrasonography, which is used as the gold standard for the diagnosis of atherosclerosis [26]. Because of the retrospective design of this present study, doppler ultrasonographic screening could not be used as a reference. Another potential limitation is what seems as the relatively small population that could have led to overfitting. To tackle this potential limitation, we performed structural optimization, applied additional networks, and used augmentation. Furthermore, a sample size determination analysis showed that the sample size is sufficient. A possible additional limitation may be that the data does not representative of the overall population, mostly due to the relatively high proportions of the elderly (which are in any case more susceptible to strokes), or the fact that the method of diagnosing carotid artery calcification relies on expert diagnosis and not on other laboratory examinations. In addition, Error analysis revealed that at times, CNN failed to differentiate the triticeous from the CAC (of note, the two structures are very similar), leading to lower success rates. Adding additional triticeous images to the database may contribute to a better classification and differentiate between triticeous and CAC, leading to improved performance.

We intend to conduct further research that will compare machine diagnosis to carotid ultrasound and angio-CT. We anticipate that a larger sample would improve this parameter. However, this study has significant strengths and benefits, including good AI performance, resulting in high recall (0.87) and substantial specificity (0.97), the ability to assess the algorithms’ performance for a patient, rather than just a corner, the repeatability of the results using different types of neural networks and structure. Above all, this study has the potential to provide clinics and healthcare organizations with a non-invasive, efficient, and applicable solution for the early detection of carotid calcification, both on a patient level and throughout healthcare systems.

In summary, this study shows the potential and feasibility of applying deep learning-based methods in an actual “real-world” application of automatic screening for CAC in standard panoramic dental X-rays. Applying this approach may significantly contribute to quality of life of populations and save many lives.

## Supporting information

S1 Table Sequence of layers in the CNN used to predict CAC. (XLSX)

S1 Text Sample size sufficiency analysis. (DOCX)

S1 Fig Sample size assessment: F1 score as a function of the sample size, based on random subsampling of 40%-80% of the original data. (TIF)

S2 Fig Figures A-D are panoramic CAC Corners. The yellow arrows point toward the plaque location.
(TIF)

S3 Fig Figures E and F are panoramic corners with Triticeous Cartilage calcification. The blue arrows point toward the calcification. Figures G and H are clean normal corners.
(TIF)

S4 Fig MapGrad of calcified corners. (TIF)

S5 Fig MapGrad of clean corners. (TIF)
