# Supplementary material for: Deep convolution neural network for screening carotid calcification in dental panoramic radiographs
Source: PLOS Digit Health. 2023 Apr 12;2(4):e0000081. doi: 10.1371/journal.pdig.0000081 (PMC10096511; doi:10.1371/journal.pdig.0000081)
Supplement: S1 Text — (DOCX) [file pdig.0000081.s002.docx]

**Text S1 - Sample size sufficiency analysis**

In order to determine whether the sample size is sufficient for this study, we used random subsamples of different sizes, and repeated the main analysis for each subsample. Results show that performance has barely changed when using a subsample of 80% of the original database, emphasizing that the current sample size is sufficient for this study. See Supplementary Figure S1.
