# Supplementary figures and images for: Deep convolution neural network for screening carotid calcification in dental panoramic radiographs
Source: PLOS Digit Health. 2023 Apr 12;2(4):e0000081. doi: 10.1371/journal.pdig.0000081 (PMC10096511; doi:10.1371/journal.pdig.0000081)

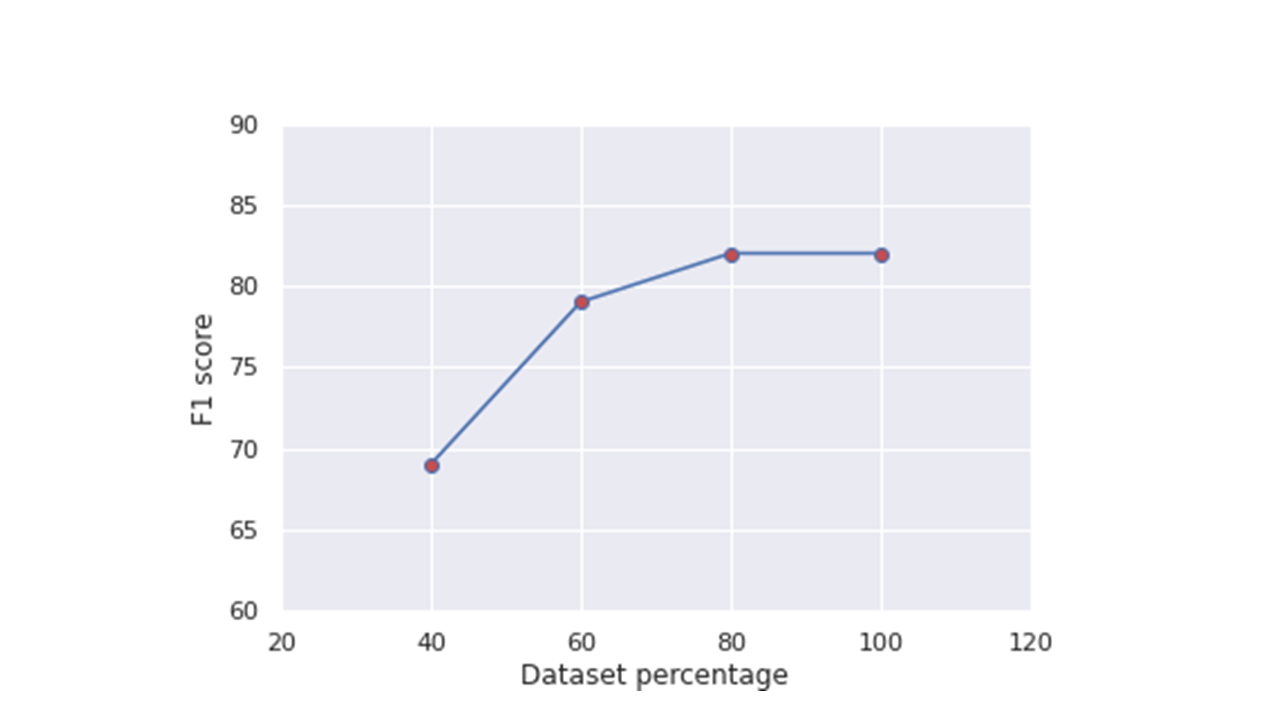

Supplement: S1 Fig — (TIF) [file pdig.0000081.s003.tif]

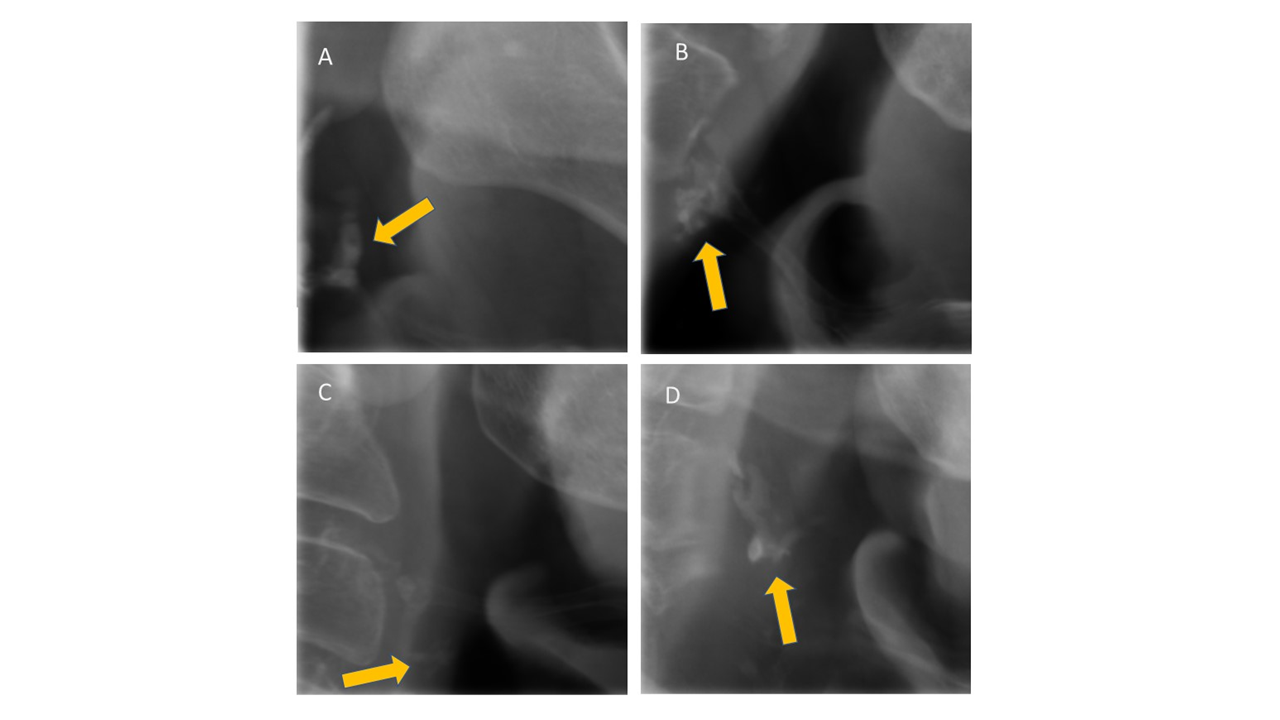

Supplement: S2 Fig — The yellow arrows point toward the plaque location. (TIF) [file pdig.0000081.s004.tif]

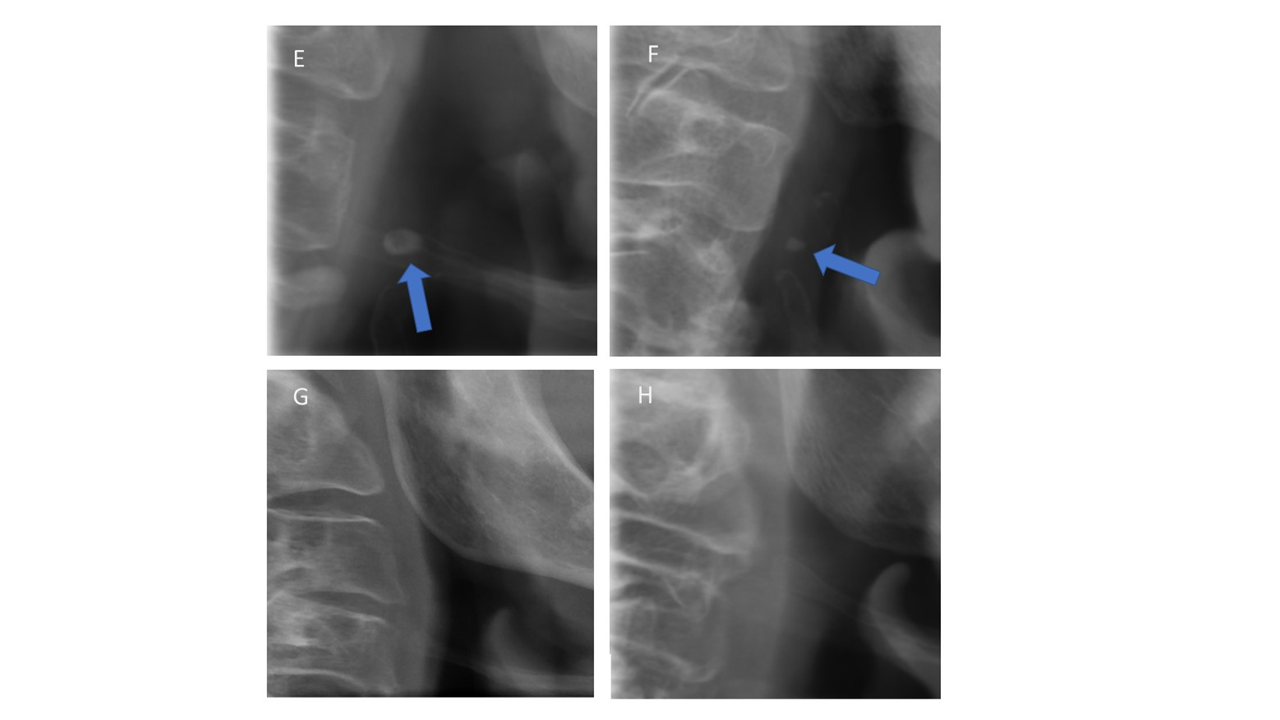

Supplement: S3 Fig — The blue arrows point toward the calcification. Figures G and H are clean normal corners. (TIF) [file pdig.0000081.s005.tif]

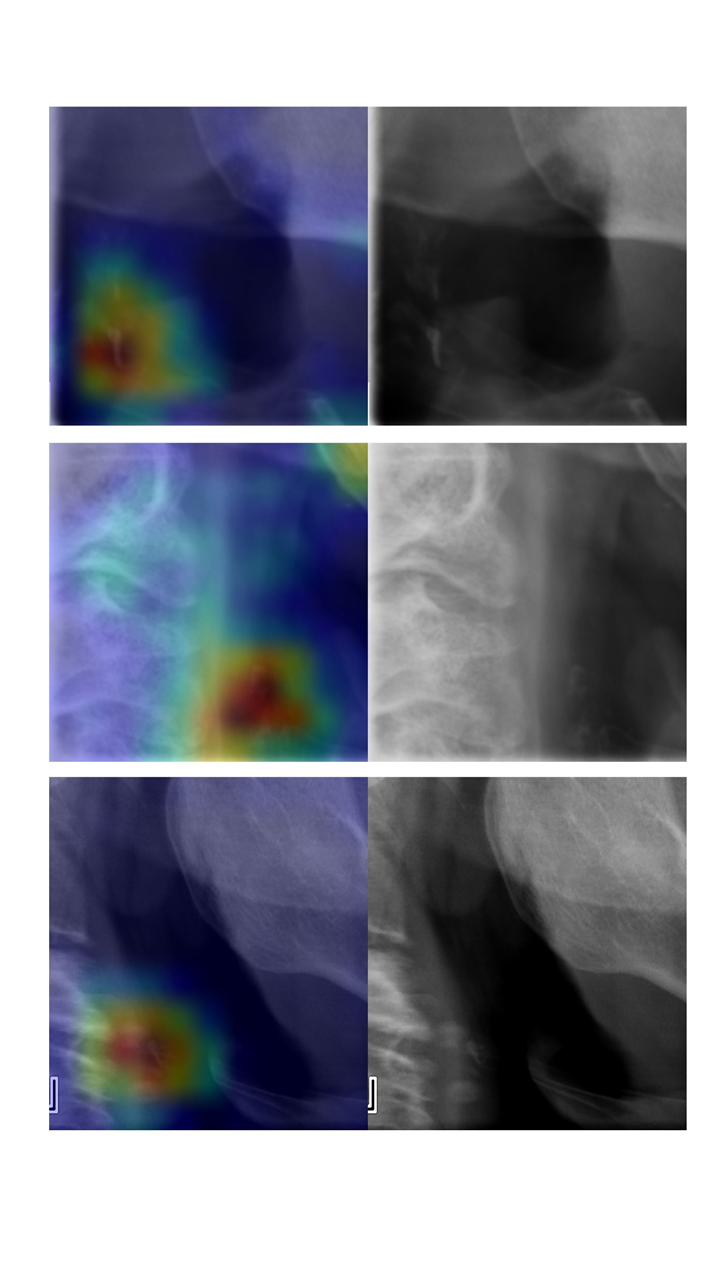

Supplement: S4 Fig — (TIF) [file pdig.0000081.s006.tif]

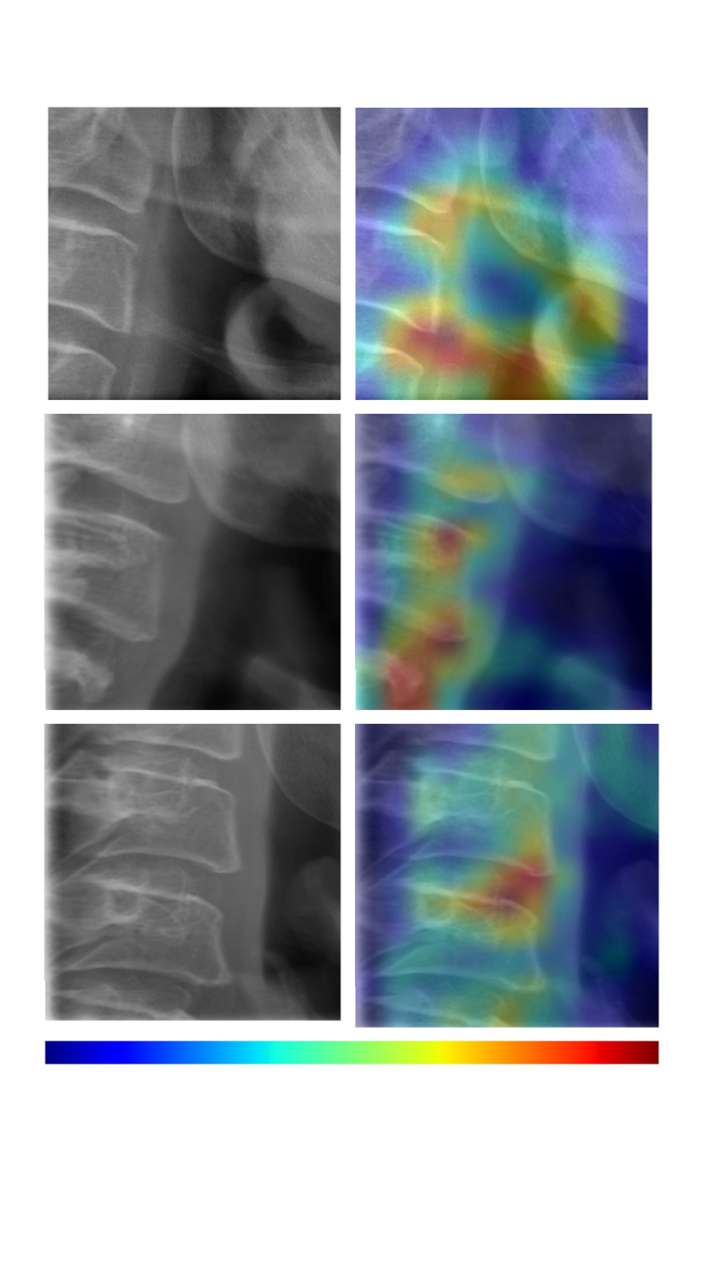

Supplement: S5 Fig — (TIF) [file pdig.0000081.s007.tif]
